# Adverse events in spine surgery: a prospective analysis at a large tertiary center in Germany

**DOI:** 10.1007/s00701-023-05752-x

**Published:** 2023-08-09

**Authors:** Pavlina Lenga, Philip Dao Trong, Vassilios Papakonstantinou, Karl Kiening, Andreas W. Unterberg, Basem Ishak

**Affiliations:** Department of Neurosurgery, Heidelberg University Hospital, Im Neuenheimer Feld 400, 69120 Heidelberg, Germany

**Keywords:** Adverse events, Dural leakage, Spinal surgery, Tertiary care, Wound infection

## Abstract

**Study design:**

Prospective study

**Objectives:**

The occurrence of adverse events (AEs) during surgery is a major cause of increased economic costs, disability, or even death. This study aimed to prospectively identify and quantify AEs in patients undergoing spinal surgery at a neurosurgical tertiary care hospital.

**Methods:**

Patients who underwent spinal surgery and were discharged between January 2019 and December 2022 were enrolled prospectively. Each patient underwent a peer-reviewed AE evaluation at discharge. An AE was defined as any event that occurred up to 30 days postoperatively and resulted in an undesirable outcome. Patients were allocated to four groups according to spinal pathology (degenerative, oncologic, traumatic, and infectious).

**Results:**

During the study period, 1778 patients with a mean age of 55.4 ± 10.5 years underwent surgery. Elective surgery was performed in 90.8% (1615/1778) of patients, while emergency surgery was performed in 9.2% (163/1778). The overall rate of surgery-related AEs was relatively low (8.7%). Degenerative pathologies were the most frequent reasons for surgery (78.5%, 1396/1778). Wound infection was the most prevalent AE in patients with degenerative diseases (1.4%), of which 1.1% required revision surgery. Wound infection, dural leakage, and new neurological deficits had the same prevalence (2.1%) in patients with spinal tumors. Among patients with spinal trauma, two presented with postoperative epidural bleeding and underwent emergency surgery. Postoperative wound infection was the most prevalent AE in this group (9.5%), with 7.0% of affected patients requiring revision surgery. The overall rate of non-surgery-related AEs was 4.3%, and the overall mortality rate was low (0.4%).

**Conclusion:**

AEs in spinal surgery remained low, with a prevalence of 8.7%. Documentation of AEs as part of clinical routine may be a key tool for identifying the occurrence of surgery-related and non-surgery-related AEs.

## Introduction

In modern healthcare delivery models, the occurrence of adverse events (AEs) during surgery is a major cause of increasing economic costs, disability, and even death [15, 18]. Therefore, to identify complications and their underlying mechanisms, morbidity and mortality conferences (MMC) have emerged in the current context, aimed at preserving or preventing repeated presentations [14]. Such mechanisms are paramount in neurosurgery, where potential AEs are cost-intensive and can lead to severe patient harm such as reoperation, transfer to an intensive care unit (ICU), postoperative neurological deficits, or even death [13, 21, 24]. However, current evidence is overwhelmingly based on retrospective studies, and their incidence remains unknown. A recent study from our group highlighted the importance of sufficient documentation of AEs after neurosurgical procedures as the basis for effective quality control in modern-day hospital management [5].

The potential array of adverse events that may ensue following spinal surgery is broad and markedly contingent upon the specific type of operation. Decompressive surgeries, including laminectomy and discectomy, carry inherent risks such as dural tears, nerve damage, and postoperative hematoma [9, 18]. More intricate operations, such as spinal fusion, present an augmented risk profile encompassing hardware malfunction, non-union, infection, and adjacent segment disease [9]. Even minimally invasive spinal surgeries, notwithstanding their reduced complication rates encompassing wound infection and a comparatively abbreviated hospital stay, are not devoid of adverse events. Notable among these are nerve damage and a risk of radiation exposure due to the utilization of fluoroscopy [4]. Deformity surgeries present a distinctive risk spectrum, ranging from acute issues such as significant blood loss and neurologic complications, to long-term complications such as pseudarthrosis or implant failure [19]. Moreover, surgical procedures employing an anterior approach to the cervical spine, including anterior cervical discectomy and fusion (ACDF), carry unique risks such as dysphagia, recurrent laryngeal nerve injury, and esophageal perforation [21]. As such, it is discernible that the likelihood of adverse events in spinal surgery is intrinsically tied to the complexity of the procedure itself, as well as the surgical approach and the specific spinal region under treatment. Furthermore, the risk profile for adverse events in spinal surgeries differs significantly, depending primarily on the complexity of the operation and the patients’ characteristics. Simple procedures such as microdiscectomies or laminectomies often entail lower complication rates due to their minimally invasive nature and the generally healthier patient demographic [14]. In contrast, complex surgeries like multilevel fusion or deformity correction are associated with a higher risk profile, attributed to increased operative demands and a patient population often burdened with more comorbidities [19]. Consequently, a comprehensive preoperative assessment and careful surgical planning become vital to mitigate these risks.

It should be emphasized that most studies, including ours, have focused on all types of neurosurgical procedures. The prevalence of spinal surgery is steadily increasing, and the evolution of its techniques and principles has led to significant advances in this field. The challenge of determining the incidence of AEs after spinal surgery, particularly regarding the related pathology, is apparent. Previous studies have reported rates ranging from 9.2 to 14.0% [5, 26]. However, obtaining accurate and reliable data on the incidence of AE in spine surgery remains challenging, as the data typically comes from either claims data based on ICD codes, which may underestimate or miss serious events, or from small retrospective case series with limited numbers of patients and follow-up data.

Although there is an abundant literature concerning adverse events in spine surgery, we perceived a gap in terms of comprehensive, prospective studies evaluating adverse events across a broad patient population undergoing diverse spinal surgeries in a tertiary care center.

## Methods

### Study design

This is a prospective study collecting data from a single-center neurosurgical tertiary care hospital. The study received approval from the ethics committee of our institution (reference S-425/2022), and it was conducted in accordance with the Declaration of Helsinki. The necessity for informed consent was eliminated in this particular situation, given that the completion of POPAE forms is part of the standard institutional procedure. It is thus integrated into the normal operational practices of our institution and does not require explicit individual consent. Our data collection and analysis followed established protocols as detailed in our prior work (Dao Trong P, et al., 2023). This protocol involves a thorough comparative analysis between the data in our prospectively compiled Post-Operative Adverse Event (POPAE) database and the hospital administration database. Further, our routine quarterly reviews help us spot and investigate statistical anomalies, ensuring the validity of our data. As previously described by our study group, 15 board-certified neurosurgeons and 18 resident neurosurgeons continuously registered and updated cases in our database [5]. Upon discharge, each patient received a postoperative adverse event (POPAE) form, which was completed by the responsible physician in the ward. Before the case was registered in our database, the supervising senior attending reviewed the form and, upon approval, the data was entered into the database. If readmission occurred within 30 days after the initial surgery, the treating team was automatically alerted. Cases with a complicated course were regularly discussed by all neurosurgical staff during MMC. For this analysis, data from at least 18 patients with spinal pathologies were extracted and analyzed, while pediatric patients were excluded.

In this study, our focus was on assessing the adverse events that transpired within the first 30 days following spinal surgery. Consequently, long-term complications or events beyond this timeframe were not included in our analysis. This early postoperative period was chosen for its critical role in immediate surgical recovery and its commonly accepted usage in assessing short-term surgical complications. In our study, we utilized a strategy of prospective data recording. As patients underwent their respective spinal surgeries and moved through their immediate recovery phase, pertinent details alongside any adverse events occurring within the first 30 days post-surgery were systematically logged into a specialized database. This real-time data capture ensured timely and precise documentation of the postoperative outcomes.

### Definitions

The following categories of AEs were established: wound event, postoperative infection, CSF fistula, malpositioning of the implanted material, new neurological deficits, rebleeding, and failure to achieve the surgical goal. Elective surgery was defined as any intervention scheduled at least 1 day in advance, while non-elective surgery included emergency procedures and revision surgery. Patients with spinal pathologies were divided into the following groups: degenerative disease, tumor, trauma, or infected spine. Patients who were allocated into the infected spine group suffered from vertebral osteomyelitis (spondylitis, discitis, spondylodiscitis or septic facet joint) and spinal epidural or intradural abscesses [30]. The categorization concerning neurosurgical adverse events has been previously described by our study group and includes AE occurring within 30 days after initial surgery [5].

## Results

### Study population and baseline characteristics

A total of 1778 patients, with a mean age of 55.4 ± 10.5 years, underwent surgery between January 2020 and December 2022. Elective surgery was performed in 90.8% (1615/1778) of patients, while emergency surgery was performed in 9.2% (163/1778). The overall rate of surgery-related AEs was relatively low (8.7%). Degenerative pathologies were the most common reason for surgery (78.5%, 1396/1778). A detailed description of the study population is presented in Table 1.

**Table 1:** Baseline characteristics

	*N* = 1778	%
Age, years (mean, SD)		
Sex (*n*, %)		
Male	960	54.0
Female	818	46.0
Non-Elective	163	9.2
Elective	1615	90.8
Spinal		
Degenerative	1396	78.5
Tumor	234	13.2
Trauma	47	2.6
Infection	101	5.7

*SD*, standard deviation

### Occurrence of surgery-related AEs

#### Degenerative disease

The most prevalent AE in patients with degenerative diseases was wound infection (1.4%), of which 1.1% required revision surgery. Notably, new neurological deficits and dural leaks were present in 1.1% and 1.0% of patients, respectively. All cases with dural leaks were surgically revised, while only three cases underwent a second-look surgery to determine the cause of neurological worsening. The mortality rate was low (0.07%). Two patients aged 82 years died due to pulmonary embolism after cemented augmented posterior fusion surgery, while one patient aged 79 years died due to postoperative heart failure.

#### Tumor disease

Wound infection, dural leaks, and new neurological deficits presented with the same prevalence of 2.1%. Revision surgery was necessary in all cases with wound infection and dural leaks, and only in three cases with new neurological deficits. The incidence of a second transfer to the ICU was 6.8%, which was attributable to renal dysfunction or cardiac disease.

#### Trauma

Among the patients with spinal trauma, two presented with postoperative epidural rebleeding, and emergency surgery had to be performed. Wound infection, postoperative infection, malpositioning of the implanted material, and neurological deficits were present in only one case, respectively. Of these patients, 12.8% were transferred to the ICU due to non-surgery-related complications.

#### Infected spine

Postoperative wound infection was the most prevalent AE in this group (9.5%), and 7.0% of affected patients required revision surgery. Dural leaks were present in 3.5% of patients, all of whom underwent revision surgery. The mortality rate was low (1.0%).

A detailed breakdown of surgery-related AEs associated with spinal pathology is shown in Table 2.

**Table 2:** Summary of surgery-related adverse events

	*n*	%	Revision surgery	%
Wound event				
Degenerative	20	1.4	16	1.1
Tumor	5	2.1	5	2.1
Trauma	1	2.1	1	2.1
Infection	19	9.5	14	7.0
Dural leak				
Degenerative	14	1.0	14	1.0
Tumor	5	2.1	5	2.1
Trauma	1	2.1	1	2.1
Infection	7	3.5	7	3.5
Postoperative infection				
Degenerative	8	0.6	4	0.3
Tumor	2	0.9	2	0.9
Trauma	1	2.1	1	2.1
Infection	1	0.5	1	0.5
Malposition of implanted material				
Degenerative	6	0.4	6	0.4
Tumor	1	0.4	1	0.4
Trauma	1	2.1	1	2.1
Infection	1	0.5	0	0.0
New neurological deficits				
Degenerative	15	1.1	3	0.2
Tumor	5	2.1	1	0.4
Trauma	1	2.1	0	0.0
Infection	2	1.0	1	0.5
Rebleeding				
Degenerative	14	1.0	7	0.5
Tumor	1	0.4	1	0.4
Trauma	2	4.2	2	4.2
Infection	1	0.5	0	0.0
Surgical goal not achieved				
Degenerative	10	0.8	6	0.4
Tumor	3	1.3	1	0.4
Trauma	0	0.0	0	0.0
Infection	0	0.0	0	0.0
Others				
Degenerative	6	0.4	3	0.2
Tumor	0	0.0	0	0.0
Trauma	0	0.0	0	0.0
Infection	1	0.5	0	0.0
Secondary transfer to IMC or ICU				
Degenerative	14	1.0	––	––
Tumor	16	6.8	––	––
Trauma	6	12.8	––	––
Infection	13	6.5	––	––
Death				
Degenerative	3	0.07	––	––
Tumor	2	0.9	––	––
Trauma	1	2.1	––	––
Infection	2	1.0	––	––

### Occurrence of non-surgery-related AEs

The overall rate of non-surgery-related AEs was 4.3%. Urinary tract infection was the most prevalent AE in all groups (1.6%, 28/1778), followed by acute renal failure (0.5%, 10/1778) and pneumonia (8/1778, 0.4%). A detailed breakdown by spinal pathology is presented in Table 3.

**Table 3:** Non-surgery-related adverse events by spinal pathology

	*n*	%
Degenerative (*n*, %)	1396	
Acute renal failure	4	0.3
Respiratory deficiency	1	0.07
Heart failure	4	0.3
Pneumonia	4	0.3
Pulmonary embolism	1	0.07
Urinary tract infection	22	1.6
Tumor (*n*, %)	234	
Acute renal failure	3	1.3
Respiratory deficiency	1	0.4
Heart failure	0	0.0
Pneumonia	2	0.9
Pulmonary embolism	0	0.0
Urinary tract infection	4	1.7
Trauma (*n*, %)	47	
Acute renal failure	1	0.4
Respiratory deficiency	0	0.0
Heart failure	1	0.4
Pneumonia	1	0.4
Pulmonary embolism	1	0.4
Urinary tract infection	1	0.4
Infection (*n*, %)	101	
Acute renal failure	2	2.0
Respiratory deficiency	1	0.9
Heart failure	2	2.0
Pneumonia	1	0.9
Pulmonary embolism	0	0.0
Urinary tract infection	1	0.9

## Discussion

To the best of our knowledge, this study explicitly reports on AEs after spinal surgery with respect to different spinal pathologies based on a large, prospectively compiled database. We found that the overall rate of AEs was relatively low at 8.7%, whereas revision surgery was necessary in 5.8% of cases, with wound infection being the most prevalent AE across all groups. Secondary transfer to the ICU or IMC was mainly indicative of patients experiencing trauma, followed by tumor and infection; only 14 patients needed ICU monitoring after treatment. It should be emphasized that the overall mortality rate remained low at 0.4%, and the cause of death was not related to the spinal procedure but was due to heart failure or respiratory insufficiency. In addition, the rate of non-surgery-related AEs was relatively low (4.1%), which was indicative of a safe and thorough postoperative care.

We acknowledge that adverse event rates and types can indeed vary depending on the specific region of the spine involved. For instance, Goel et al. undertook an in-depth analysis of complications arising from craniovertebral junction surgeries, revealing that certain adverse events tend to occur more frequently in this particular region [11]. Goel’s work illuminates the complexity inherent in this region, underlining the need for specialized knowledge and careful surgical planning to mitigate these risks. Similarly, a comprehensive study by Nanda et al. (2014) investigated the complications associated with anterior cervical diskectomy and fusion for cervical degenerative disk disease. They found a unique profile of surgical complications, pointing to the necessity of taking a tailored approach to prevent and manage potential adverse events in cervical procedures [22]. Hassanzadeh et al. (2013) carried out a study examining adverse outcomes in the context of spinal deformity treatment in adults 60 years old and older [12]. They specifically focused on three-column osteotomies, revealing distinct risk patterns associated with these thoracic and lumbar procedures [12]. Furthermore, Rihn et al. performed an analysis of patients with lumbar disc herniation and highlighted the impact of symptom duration on treatment outcomes [27]. Their work adds another layer of complexity to understanding adverse events, emphasizing the interplay between preoperative factors and surgical outcomes. Adding an economic dimension to the discussion, Martin et al. reported on the significant variability in hospital costs and trends related to lumbar fusion procedures in the USA over a decade [19]. This variation underscores the economic implications of adverse events and the potential benefits of targeted efforts to reduce complication rates in this commonly performed procedure. Taken together, these studies serve to underscore the multifaceted nature of adverse events in spinal surgeries. Each region of the spine presents its unique challenges, complications, and considerations. While our study casts a wide net to provide a broad understanding, we recognize that future research, focusing on region-specific outcomes, can offer more granular insights that could further optimize patient care and safety in spinal surgeries.

Our analysis indicates that approximately 9% of the spinal surgeries we conducted fell under emergency procedures. This percentage, when juxtaposed with data from other centers, provides an intriguing point for discussion. Within the literature, there is a wide range in the incidence of emergency spinal surgeries. Smith et al. reported a proportion of 15% [18], while the study by Jones et al. (2021) found a lower rate of 7%. Several factors contribute to these variations, including differing patient demographics, clinical specialties, and healthcare delivery systems. For example, institutions that mainly serve patients with a higher risk for spinal emergencies, such as trauma or tumor cases, might record a higher percentage of emergency procedures [18]. In contrast, centers focusing on elective surgeries for degenerative spine conditions may report a lower rate [15].Furthermore, regional differences in healthcare delivery and policy can also influence these rates. In areas with well-established referral systems and policies that centralize emergency care to certain hospitals, the incidence of emergency spinal surgeries could be higher. Conversely, in regions with more dispersed healthcare systems, the rate could be lower. Thus, when interpreting our rate of emergency spinal surgeries, these multiple factors must be taken into account. Our findings, though specific to our context, contribute to the broader understanding of how healthcare delivery models, patient demographics, and clinical focus can influence the incidence of emergency spinal surgeries. This underscores the need for future research that explores these contributing factors in more detail. Such studies could lead to improvements in healthcare systems and patient management, particularly in optimizing the distribution of emergency versus elective spinal surgeries.

Our study reported thromboembolic events in just two patients, representing a relatively low incidence compared to some published studies. For instance, a large-scale study by Sebastian et al. (2021) examining 43,777 patients who underwent thoracolumbar surgery reported a 2.2% incidence of VTE [17]. This study identified several risk factors for VTE, including older age, higher body mass index (BMI), and a prior history of VTE [17]. Interestingly, they also discovered that patients undergoing revision surgery had a higher risk of developing VTE, highlighting the significance of surgical complexity and patient history in predicting adverse events. Our lower incidence of thromboembolic events might be attributed to the particular nature of our study, including the patient demographics and the surgical procedures performed. We acknowledge that our research might not fully encompass the potential diversity and complexity seen in broader spine surgery populations. We must also consider the possibility of underreporting of thromboembolic events in our study. Despite our proactive data collection, some events may have been missed, particularly if they occurred after patients’ discharge or were asymptomatic. The lessons from Yoshioka and Sebastian’s studies underscore the importance of implementing robust systems to capture and monitor postoperative complications, including VTE, to ensure the safety and well-being of patients.

To date, several protocols have been developed for the universal evaluation of spinal AEs. Rampersaud et al. evaluated the inter-and intra-observer variability of the Spinal Adverse Events Severity System (SAVES-V2), which is a classification system with 14 intra-operative and 22 postoperative AEs with a grade range of 1 to 6 (6 indicating death) [25]. They reported that SAVES-V2 demonstrated a substantial intra- and inter-observer agreement regarding AE severity for spinal trauma cases, and a perfect agreement for degenerative cases. This finding indicates that spinal trauma is a more complex scenario due to patients presenting with a wide spectrum of spine- and non-spine-related injuries, leading to greater variation in postoperative care (normal ward or ICU) compared to typical elective scenarios presented by patients with degenerative diseases [25]. SAVE-2 appears to be an easy-to-use tool that consistently captures AEs caused by spinal surgery. However, the study had certain limitations. Although experienced surgeons evaluated the reliability of the proposed classification system, and even excluded complex cases such as spinal tumors or infections, only a fair agreement was reached. Therefore, it could be argued that the observed reliability might be even lower if AEs were to be evaluated by residents. Although the classification system has been tested as part of a prospective clinical multicenter trial, it has not yet become the standard for clinical routine at the institution, and its utilization remains challenging. Furthermore, the AE rates were extracted from patients with traumatic and degenerative pathologies, while tumor- or infection-related events were excluded; as the latter might present with a more complicated disease course, this could lead to further disagreement. In contrast, in the present study, a postoperative AE form was routinely provided to patients upon discharge, which was filled out by the responsible neurosurgical physician and reviewed by the supervising senior attending physician, and only approved data were entered into the institutional database. Furthermore, complex cases were regularly discussed in a conference with all physicians in the neurosurgical department to identify potential deficits in care that could lead to the occurrence of AEs, which aimed to prevent them in the future. To date, a more standardized classification system (Clavien–Dindo Classification) has been deployed to our institutional algorithm, which would enhance the documentation of our data as well as enable better comparability across neurosurgical centers [7, 8, 16].

The rate of comorbidities should also be considered when opting patients to a surgical procedure. Ayling et al. reported AE rates of 2.4% and minor AE rates of almost 1.9% based on claims data from a retrospective study of 3533 patients who underwent lumbar spine surgery for the treatment of degenerative pathologies [3]. A previous study demonstrated a wide range of AE rates after lumbar spine surgery, ranging from 3.7 to 12.8%; however, the definition of AE was not clearly stated [6]. In contrast to these studies, our findings showed AE prevalence of 6.7%. The primary difference between the studies was that our data were obtained from a prospectively compiled database, and each case was evaluated by a team of trained physicians rather than relying solely on ICD-10 codes. The discrepancy in prevalence based on the evaluation technique was also highlighted by Street et al., who found a much higher prevalence of AEs (1.7 times higher) in patients undergoing surgery for traumatic spinal cord injuries when the data were analyzed by the SAVE system rather than ICD-10 codes [29]. This indicates that if ICD-10 code data were used alone, the incidence of AEs would be substantially underestimated, leading to an underestimation of disease complexity as well as contribute to the enormous economic and medical burden associated with their acute care, a phenomenon recognized and carefully analyzed by Nasser et al. [23].

Cost-effectiveness represents a critical pillar for patient care; hence, a meticulous documentation of AEs is critical for the evaluation of patient outcome. Herein, it should be emphasized that the impact of AEs on costs of spinal surgery is substantial, and a deeper understanding of their consequences is unambiguously warranted to optimize patient-reported outcomes. For instance, in a recent review metanalysis with data from USA and Canada, the treatment costs due to AEs caused mainly by infection ranged from $15,819 to $38,701, while the overall costs due to the occurrence of AEs were 2.3 to 3.1 times greater compared to patients without complications [32]. In Germany, the health care costs are also on the rise. According to the Federal Statistical Office of Germany, they have almost been doubled in the past 20 years from €2724 per capita and year in 2001 to €5298 in 2021. Especially, concerning spine diseases, the hospital costs have tremendously increased from €340 Mill. in 2015 to €420 Mill. in 2020 [1]. However, a closer analysis examining reasons leading to such a substantial increase in costs is unreassuringly still understudied. Therefore, it is crucial to quantify the cost of specific complications associated with spine surgery so that health care providers can optimize patient outcomes and control escalating health care costs associated with management of AEs.

Our study prospectively identified and quantified adverse events (AEs) in patients who underwent spinal surgery at a neurosurgical tertiary care hospital. The observed prevalence of AEs was 8.7%, with wound infections and dural leakage being the most frequent. Notably, the overall mortality rate was relatively low at 0.4%. Sarthein et al., in their monocentric study of AEs in neurosurgery, reported on approximately 633 patients undergoing spinal surgery, a number much lower than our present cohort [28]. The researchers themselves stated that their primary focus was on cranial surgery, so complications specific to spinal surgery were not explicitly reported. Consequently, a direct comparison with our findings could not be made. Interestingly, a similar pattern of AEs was seen in the prospective study by Lovi et al. [17]. They evaluated a cohort of 364 pediatric and adult patients and found dural leakage and neuropathic pain to be quite prevalent post-surgery, mirroring our findings. However, their overall AE prevalence was nearly 21.0%, significantly higher than our reported 7.8%. This disparity could potentially be attributed to their inclusion of pediatric patients undergoing surgery, a factor that may not accurately reflect the real-world prevalence of AEs. Furthermore, their study exclusively focused on fusion lumbar and deformity surgery, procedures associated with increased risks compared to common surgeries like microdiscectomy and laminectomies. The Spine Tango Registry, which includes data from 52 centers in 18 countries, noted that surgeries due to degenerative diseases were more prevalent [20]. Interestingly, they reported a lower prevalence of dural leakage than our study. This discrepancy may be due to the low coverage of documented procedures within departments, an issue that becomes even more pronounced at the national or international scale. Our study, like Spine Tango, likely suffers from a reporting bias due to low participation rates and cautious reporting of complications, given the voluntary nature of participation. The improvement of results’ representativeness and participation rates could be achieved by making documentation mandatory, enforcing binding rules, and implementing monitoring mechanisms. While the Spine Tango registry serves as a valuable source of data for spinal surgery outcomes, our study presents several key strengths that potentially address its limitations. One significant advantage is our application of standardized methods across all aspects of data collection and analysis. This uniform approach aids in creating a more consistent, comparable, and interpretable dataset, which is often challenging in large, diverse registries like Spine Tango. Our mandatory inclusion of every patient ensures our data accurately represents the full range of surgical outcomes, providing a more comprehensive and representative analysis of AEs in spinal surgery.

### Limitations

While this study is the first to describe AEs based on a prospectively compiled database of patients with all types of spinal pathologies, it has some limitations. First, due to the short-term follow-up of 30 days, we could not identify long-term complications, such as adjacent-level disease after spinal fusion surgery. Second, despite thorough supervision of each case, misinterpretation of events may have occurred. Third, applying a systematic analysis such as the SAVE-V2 classification system might reduce potential inter- and intra-observer variability. Furthermore, the effects of education on MMC could not be objectively determined. Even though our study mandates participation for every patient, there could be potential bias related to patient selection for surgery or the decision-making processes leading to different surgical procedures. Lastly, our study does not account for potential confounding factors such as socio-economic status, comorbidities, or lifestyle factors, which might impact both the occurrence and reporting of AEs. An important point to consider is the scope of surgical procedures performed at our institution. Our findings do not encompass experiences related to minimally invasive spine surgery, as these procedures are not part of our current surgical repertoire. Minimally invasive techniques, which often result in fewer complications and quicker recovery times, may present a different profile of AEs. The absence of such data potentially limits the comprehensiveness and applicability of our findings to the wider landscape of spinal surgery. Furthermore, our institution does not undertake deformity surgery. These complex procedures, which involve the correction of severe spinal curvature, carry a unique and often elevated risk profile. Consequently, our findings may not fully represent the breadth of AEs associated with the entire spectrum of spinal surgery. Thus, while our study provides important insights, the generalizability of our findings to all spinal surgery settings, particularly those employing minimally invasive techniques or performing deformity surgeries, is constrained. Future research, including a wider variety of surgical techniques and pathologies, will be essential for a more complete understanding of AEs in spinal surgery.

### Outlook

Preoperative assessment serves as the first line of defense in managing postoperative complications. According to Carreon et al., identifying and addressing modifiable risk factors such as obesity, malnutrition, and diabetes in the preoperative phase is critical [4]. By effectively managing these conditions, surgeons can improve patients’ physiological status, thereby reducing the risk of postoperative complications. This approach is further supported by Arozullah et al., who developed a predictive model for postoperative complications based on preoperative variables [2]. Their model suggests that optimizing preoperative conditions can decrease the risk of complications, reinforcing the idea that a patient’s preoperative status plays a pivotal role in postoperative outcomes. The surgery itself is a critical phase where complications can be mitigated. Ghobrial et al. elucidate how the right surgical technique and approach, like the use of intraoperative neuromonitoring, can significantly decrease neurological adverse events [10]. Their findings imply that the consideration of meticulous surgical approaches and advanced techniques can result in safer surgeries and better postoperative outcomes. Further adding to this perspective, Fehlings et al. (2012) found that early surgical intervention can reduce complications in patients with traumatic spinal cord injury [9]. This suggests that the timing of surgical intervention, carefully determined on a case-by-case basis, can also influence the risk of adverse events. In the postoperative phase, standardizing care protocols based on evidence-based practices has been proven beneficial. Wick et al. underscore this point by outlining an evidence-based care pathway to reduce postoperative complications [31]. This approach includes proper wound care, pain management, early mobilization, and vigilant monitoring for early signs of complications, highlighting that structured postoperative care can significantly improve patient outcomes. In conclusion, our study emphasizes that to ensure patient safety and improve outcomes in spinal surgery, a comprehensive approach spanning preoperative, intraoperative, and postoperative phases is crucial. Preventive strategies should be considered and implemented diligently, with an aim to cater to the unique needs of each patient.

## Conclusion

AEs in spinal surgery are a frequent phenomenon with a prevalence of 8.7%, and procedures should be established in clinical practice to identify them early and avoid repetition. In this study, the mortality rates remained low at 0.4%; thus, AE documentation as part of clinical routine may be a key tool for identifying the occurrence of surgery-related and non-surgery-related AEs. Prospective data on the incidence of all types of AEs in spine surgery can enhance not only the education of our patients but also the discussion of quality-based accreditation and reimbursement systems in upcoming healthcare reforms.

## Data Availability

The datasets generated during and/or analyzed during the current study are available from the corresponding author on reasonable request.

## Code availability

The code supporting the findings of this study is available from the corresponding author upon reasonable request.
